# Maternity behind and beyond bars: analysis from the perspective of protection bioethics

**DOI:** 10.1590/0034-7167-2022-0576

**Published:** 2025-01-10

**Authors:** Denise Santana Silva dos Santos, Dulcinéia Ghizoni Schneider, Mara Ambrosina de Oliveira Vargas

**Affiliations:** I Universidade do Estado da Bahia. Salvador, Bahia, Brazil; II Universidade Federal de Santa Catarina. Florianópolis, Santa Catarina, Brazil

**Keywords:** Prisons, Maternity, Bioethics of Protection, Vulnerability, Women, Cárceles, Maternidad, Bioética de la Protección, Vulnerabilidad, Mujeres

## Abstract

**Objective:**

to analyze how motherhood is expressed in female prison units from the perspective of Bioethics of Protection.

**Method:**

qualitative research with an ethnographic approach, developed in two women’s prison units. Participantes were: six mothers deprived of liberty, 15 health professionals, and nine prison officers. For data collection, semi-structured interviews and descriptive observation were used. Data analysis was based on the Content Analysis technique, thematic category.

**Results:**

three categories emerged: women and children violated behind bars (inequities); mothers and children in prison exacerbating imbalances, tensions and conflicts; and limits and references for resocialization.

**Final Considerations:**

the Bioethics of Protection proposal appears as a valid tool for the analytical direction of the process of confronting issues in the scope of public health in prison units, considering vulnerable groups and aiming at equity and human dignity.

## RESUMEN

**Objetivo:**

analizar cómo se expresa la maternidad en las cárceles de mujeres desde la perspectiva de la Bioética de la Protección.

**Método:**

se trata de una investigación cualitativa con enfoque etnográfico, llevada a cabo en dos unidades penitenciarias femeninas. Participaron en el estudio seis madres reclusas, quince profesionales sanitarios y nueve agentes penitenciarios. Los datos se recogieron mediante entrevistas semiestructuradas y observación descriptiva y se examinaron con la técnica de Análisis de Contenido por categorías temáticas.

**Resultados:**

surgieron tres categorías: mujeres y niños vulnerables entre rejas (iniquidades); madres e hijos en prisión, con desequilibrios, tensiones y conflictos exacerbados; y límites y referencias para la resocialización.

**Consideraciones Finales:**

la propuesta de la Bioética de la Protección surge como una herramienta válida para la dirección analítica del proceso de enfrentamiento de las cuestiones de salud pública en las prisiones, a favor de la equidad y la dignidad humana de los grupos vulnerables.

## INTRODUCTION

The number of women in prison has increased throughout the world in recent decades, with Brazil in third place in the world ranking with 773,151 thousand people incarcerated and 37.2 thousand are women. In the mapping carried out by the National Penitentiary Department (DEPEN), in the Brazilian prison system in March 2020, of the total number of women prisoners, 12,821 were mothers of children up to 12 years old and 208 were pregnant^(1)^. This fact has an impact on the number of women who experience motherhood in the context of the prison system and, consequently, increases the number of children born in prison, making them a vulnerable population^(2)^.

The National Policy for Attention to Women in Situations of Deprivation of Liberty and Those Leaving the Prison System establishes minimum conditions of assistance to imprisoned mothers and newborns, since laws and public policies are of utmost relevance to minimize the high rates of maternal and child morbidity and mortality^(3)^. However, the study showed that although there are public policies aimed at incarcerated women, they are still far from having their rights guaranteed, respected and guaranteed by law^(4)^.

These incarcerated women/pregnant women/mothers face situations of tension, suffering, violence, insecurity, fear, anxiety and impotence in the face of the duty to protect the child, limited socio-affective support, as well as the uncertainty of the impact of the prison environment on the child’s life, since institutions do not have the infrastructure to meet the needs of the binomial^(5,6)^.

It is also important to highlight the exposure to vulnerable health conditions and frailty, compromising the health of the mother-child binomial and triggering complications such as premature births and low birth weight, and, thus, increasing maternal and neonatal morbidity and mortality rates^(7)^.

Despite the existence of studies that portray the daily life of female incarceration, there is a gap, arising from field research that deals with motherhood in prison from the perspective of Bioethics of Protection.

In this way, upon reflection on the concepts brought by Bioethics of Protection, a proposal formulated by Latin American researchers can highlight contents that arise from ethics and are related to the moral problems involved in human vulnerability, damages and concrete needs^(8)^. Therefore, Bioethics of Protection was used as a guiding instrument to evaluate the moral conflicts that permeate the experience of motherhood in prison. From this perspective, this study adopted the concept of vulnerable people described by Schramm^(8,9)^ as people who are not capable, for some reason independent of their will, of defending themselves due to the unfavorable conditions in which they live or due to the abandonment of current institutions that do not provide them with protection. They offer the necessary support to face their affected condition and try to get out of it.

Therefore, the relevance of this study lies in reflecting on the vulnerable situation experienced by women during motherhood in the prison system. Motherhood is understood as a social phenomenon and has different meanings according to the historical context^(4,7)^. Therefore, it is noteworthy that the conditions of pregnancy, labor and birth influence maternal and child morbidity and mortality, an indicator that impacts the level of human development in a country.

## OBJECTIVE

To analyze how motherhood is expressed in female prison units from the perspective of Bioethics of protection.

## METHODS

### Ethical aspects

This research respected national and international requirements regulated by research standards involving human beings, being submitted and approved by the Research Ethics Committee of the School of Nursing of the Federal University of Bahia. It was also authorized by the Secretariat of Penitentiary Administration and Resocialization of the State of Bahia (SEAP) and a Free and Informed Consent Form (ICF) was prepared, read and signed by the participants.

### Type of research and theoretical framework

This is a qualitative, descriptive study of an ethnographic nature. In ethnography, the social being constructs, and often maintains, their customs, traditions and beliefs in their *modus vivendi*, in order to perpetuate them with each generation^(10)^.

Ethnography has been used in international^(11-13)^ and national^(7,14)^ studies, describing the situation of the population deprived of liberty, which demonstrates the relevance of this methodology in understanding everyday life, values and cultures developed within prison units.

### Methodological procedures

#### Study Setting

The research locus was the Women’s Penal Complex of Salvador and the Women’s Prison Unit of the *Feira de Santana* Penal Complex in the state of Bahia. These units were chosen because they have a nursery to accommodate mother and child in the state of Bahia.

The initial approach to the field of study and the participants took place through an undergraduate research project and an Outreach University Projects that develop education and health actions with women and their children in the prison unit, through monthly workshops.

#### Data Source

Six mothers who experienced motherhood in deprivation of liberty participated in the study; 15 health professionals (social worker, nurse, doctor, nutritionist, psychologist and nursing technician) directly linked to the process of caring for mothers and their children in prison; and nine prison officers. Thus, totaling 30 participants.

The research inclusion criteria were: mothers who had experienced motherhood in deprivation of liberty and were serving a prison sentence; prison officers and health professionals who make up the institution’s staff and were on duty at the time of the interview.

Mothers deprived of liberty who were in the semi-open regime, or who presented cognitive changes, were excluded from the research. And prison officers and health professionals who were on medical leave, therefore away from their work activities.

The invitation to the participants was made by the first author, during the outreach activities of the prison unit project. After acceptance, the date, place and time of the interviews were scheduled, depending on the availability of the participants.

### Data collection and organization

Data collection was based on the consolidated criteria for reporting qualitative research (COREQ) and took place between the months of January and July 2019, in two stages: the first was documentary, through the analysis of medical records and legal processes of women/ collaborating mothers who were in prison units and recording data on a form; the second used semi-structured interviews and descriptive observation.

The interviews were carried out individually, in the administrative sector room of the prison units, recorded by a digital voice recorder (with the participants’ prior consent) and lasted an average of 30 minutes. Subsequently, the interviews went through the transcription process, this stage being carried out by three scholarship holders from *Grupo Crescer*, as there was a large volume of data. However, all transcripts were reviewed by the researcher.

After being transcribed, the interviews were coded with the letter to characterize the participants: mother (M), health professional (HP) and prison officer (PO). And then the number relative to the order in which they were carried out, in order to maintain the anonymity of the interviewees.

### Data Analysis

The data obtained was analyzed according to Content Analysis^(15)^, in the Thematic Analysis modality. For the analysis, the statements were cut, taking into account the frequency of themes extracted from the interviews, in order to find the main cores of meaning, whose presence gives meaning to the proposed objective. Then, a dialogue began between the material found and the theoretical assumptions chosen for the research. The information collected in the form was triangulated with data obtained through semi-structured interviews and literature references.

Thus, three thematic nuclei were identified that supported the knowledge obtained in this study: (1) Women and children abused behind bars (inequities); (2) Mothers and children in prison exacerbating imbalances, tensions and conflicts; and (3) Limits and references for resocialization.

## RESULTS

### Characterization of participants

In the sociodemographic description, the research revealed that the participants were mostly women, only one man was interviewed and the age ranging from 18 to 55 years.

Among the women deprived of liberty, it was observed that in relation to education, five women had incomplete primary education and only one had completed primary education; In relation to marital status, all were married or lived in a stable union. As for race/color, all declared themselves black (black and brown).

It was observed that among the health professionals, 11 were black and four were white, seven reported being single, seven married and one in a stable relationship. Regarding the profession: three social workers, five nurses, two nutritionists, one doctor, three psychologists and one nursing technician.

Among the prison officers, four had higher education in courses focused on education and one of the participants had a post-graduation course. Regarding the length of service in the prison system, three had less than 10 years of service, two had between 10 and 20 years and four had more than 20 years of service. And they were all competitive.

From the reading, exploration and analysis of the statements, three analytical categories emerged entitled: “Women and children vulnerable behind bars (inequities)”; “Mothers and children in prison exacerbating imbalances, tensions and conflicts” and “Limits and references for resocialization”.

#### (1) Women and children abused behind bars (inequities)

In the first category called “Women and children abused behind bars (inequities)”, the participants’ speeches described situations of violence experienced by women deprived of their liberty:

*My life was very difficult, my partner beat me a lot, I was jealous of everything, I left home and went to live with him. Many people didn’t even understand why I was still with him, he used drugs, every time he saw me talking to someone, everything was a reason for me to be beaten, if I didn’t want to have sexual relations with him, he would beat me. When I was arrested, he sent me a message that if I didn’t have an intimate encounter with him, he would kill me here in prison, I was afraid of him, I was threatened with death several times by him.* (M3)

Women in the prison system experience loneliness and the lack of a support network to care for their children.

*I never received a visit from anyone, not even from my mother, who because of my arrest took two loans and now earns very little and still takes care of my children. There are times when I think I’m going to go crazy with so much longing* [tears in my eyes] ... [silence]. (M2)

*Women are abandoned in prison, they are more faithful and do not abandon their husbands. This is a complication for the woman, as they don’t have a partner to help with the care of the child.* (HP9)

The inadequate structure of the prison environment generates situations of inequity that interfere with the provision of care to children. The participants’ speeches express this inadequacy:

*Well, here in prison the environment is bad. We gave the boy a bath in the cell, we also wash clothes in the cell and put the clothes out to hang in the courtyard.* (M1)

*The main challenge for me is the infrastructure of prison for children. Exposing them to an unhealthy environment which is harmful to their lives.* (HP10)

It was observed that the context of vulnerability experienced by the woman and her child in prison triggers the child’s illness.

*My son here had a diaper rash, as it is very hot here, he also got heat rash. Then he developed an allergy, he had difficulty breathing because of the smoke here in the pavilion, so he had to call the prison guard to take him to the doctor for evaluation, then we were taken to the emergency room so he could be nebulized.* (M6)

*Regarding the disease, here in the prison complex it is very hot in the cell and diapers are hardly made available by the administration, when the family and the Prison Ministry do not provide them, it is very complicated, then the children end up with diaper rash. All children had some kind of skin problem, such as prickly heat, itching and diaper rash.* (HP1)

It was also noted that the deprivation of social contact experienced by children who stay with their mother in the cell without interacting with other children, family members and relatives predisposes them to future psychological damage.

[…] *and another thing that also affects me is the issue that hurts, the child is innocent and is born in prison, he has no contact with toys, with colors, with other relatives, with other children, he stays here in this unhealthy environment, with women fulfilling penalties related to different things, it is a danger. She does not receive visits from other children, nor from neighbors, her grandmother rarely comes to visit, the woman is left alone with the child inside. (*PO8)

*For me, the biggest challenge of having a child, while also being deprived of liberty, is the psychological damage that child will suffer, it is impossible to get out of this hell without any consequences. Therefore, I think it is not cool for the child to be in here, they are deprived of social contact and interaction with other children.* (HP2)

#### (2) Mothers and children in prison exacerbating imbalances, tensions and conflicts

In the second category called “Mothers and children in prison exacerbating imbalances, tensions and conflicts”, the participants’ speeches revealed the conflicts experienced by women in prison and highlighted the conflictual relationship between inmates and prison officers.

*When we feel bad here, it’s a struggle, everything is very difficult, imagine for the child, because the dear ones* [prison officers] *always think we’re lying, so they don’t believe us and it takes a while for them to come, when they do come they take them to the medical center and there they give you fever medicine, ointments for diaper rash or prickly heat and that’s it.* (M1)

Another conflict highlighted by the interviewees revealed fights between the inmates themselves.

*Here in the prison there is a lot of confusion, a lot of trouble, a lot of fights between rivals. I was scared for her in here and there would be a commotion and she would get hit.* (M5)

The interviewees’ speeches revealed that the prison context is marked by tension, fear and the fear of rebellions. Situations that make the experience of motherhood in prison even more vulnerable and expose women and their children to risk situations.

*In terms of safety, our role is to remove the child from those violent groups, more agitated groups, which have a greater atmosphere of tension. So, we try to remove them from this group, away from this interaction, so that it doesn’t affect their health as a whole.* (PO8)

*My biggest difficulty is rebellion, I’m afraid of having a riot with babies inside, you know, why? If anything happens to this baby, the press, the Public Defender’s Office and everyone will blame the Security sector for the rest of their lives, saying that it was irresponsible to keep babies in there, and that’s it. Because the child is an incapable being and must be protected.* (PO4)

#### (3) Limits and references for resocialization

In the third category called “Limits and references for resocialization”, the situation of women’s recidivism in the prison system emerged in the speeches:

*This is my second time in prison, I was first arrested for drug trafficking* [8 years]*, I was on the run when I went out for a Christmas pardon and didn’t return, then I was arrested again for drug trafficking and I’ve already been sent to the penal complex.* (M4)

*So, my first entry into prison was when I was pregnant with Ester, I stayed here for 8 months and left for House Arrest for 3 years. Then, on my second arrival, I returned, I was already pregnant with Ruth in the 8th month of pregnancy, I went straight to the discipline cell* [insurance] *where I stayed for 20 days until Ruth was born.* (M5)

During the interviews, the hope of entering the job market after serving the sentence was observed, highlighting the need for the resocialization of those released from the prison system.

*It’s horrible being away from my son, I have no words to decipher what it’s like to be away from him. All I want is to go out, get a job and take care of my son.* (M4)

However, the discredit in the resocializing role of prison on the part of professionals who work in the prison system was also noticed.

*So, I don’t believe in resocialization, or anything that says in these manuals, for me, whoever does it has to pay, is he a thief, did he steal? You have to pay. But the child didn’t do anything, he was just born in this miserable place, without any living conditions. There is no mother as a reference, because our parents are our heroes and she* [child] *has nothing, she is stuck with her mother who is a marginalized woman and still put her son here.* (HP2)

*I don’t believe in people here, like how can they change? Resocialize? I do not believe this. I believe that people don’t change and that’s why children are punished along with their mothers.* (HP5)

Finally, the State’s role of protecting women and their children in custody in the prison system was highlighted.

[…] *we want to protect that innocent being, the mother’s sense of protection, we want to protect the innocent who is under the guardianship of the state, we suffer from seeing the child born in prison, from being there for so long and then being separated.* (PO8)

## DISCUSSION

The study highlighted several situations of violence experienced by mothers and their children in prison units, revealing that these women are vulnerable, that is, they are people who do not have the means that enable them to carry out their life projects, therefore, they are not capable of achieving a dignified life^(8,9)^. For this reason, they are exposed to a series of risks that have implications for their physical and mental health, as well as the health of their child.

This situation is aggravated by the inequities existing in the prison system, exemplified in a study^(6)^, which highlights the inadequacy of the physical structure, revealed through humid and hot cells, reduced hours for sunbathing, and the absence of an appropriate place for childcare.

Researches^(5,7)^ have revealed that the lack of structure in prisons, in addition to affecting the resocialization of women deprived of liberty, harms, above all, the quality of life of these people and their children. The precarious structures of prisons in relation to the specific needs of children, which range from the absence of adequate bathrooms for the child’s hygiene to the lack of resources such as diapers, clothing, ointment to prevent dermatitis, lack of adequate space for Extending children’s clothes worsens inequalities and makes the repercussions of incarceration worse for these mothers and children.

The results of this study highlight the health inequities that exist in the prison context and lead us to think about the moral issues that occur routinely and over time, giving visibility to deficiencies and social injustices that should no longer occur. These are realities demonstrated by social imbalances that determine unequal chances of access to health and other fundamental rights^(16)^.

In this sense, the social issues experienced by children of mothers deprived of liberty can also be described as social isolation, with times and days for family visits, without the opportunity to go to other places, play in a small square, interact with other children, fundamental conditions for the child’s socialization. The child in the vulnerable context of prison is confined to the cell with his mother^(17)^.

This context of vulnerability leads children to become ill, as it exposes mothers and children to infectious diseases. Studies^(17-19)^ point to high rates of infectious diseases. That is, 20% of women in prisons have infectious diseases; 26.8% say they engage in risky sexual activity with multiple partners; 11.5% have sexually transmitted diseases; high risk of HIV/AIDS, hepatitis C, as well as the human papilloma virus associated with cervical cancer; Other sexually transmitted infections that women are at greater risk of having when they enter prison are chlamydia, gonorrhea and syphilis, which are associated with sexual victimization and prostitution.

The data reveal the vulnerability that these women/pregnant women deprived of liberty are exposed to, and experience situations of risk and damage to their health, in addition to exposing the child, which increases the risk of neonatal morbidity and mortality. These women have often experienced situations of extreme vulnerability in life outside prison walls, perpetuating social exclusion and health inequities in prison^(17,20)^.

In this sense, professionals are also exposed to illness, through exposure to psychosocial risks and biological risks through contact with communicable diseases. Working conditions are not good, as the infrastructure is precarious, the environment is unhealthy and equipment and materials are scarce, which makes it difficult to carry out work effectively and safely^(18,20)^.

Other aspects addressed by the interviewees were loneliness and the lack of a support network, elements that increase the vulnerability of these women deprived of their liberty. Historically, women are abandoned in prison and experience motherhood alone. National research has highlighted several situations that promote mental disorders, including: loneliness, sadness due to abandonment by family members, fear due to inadequacy in adapting to prison culture, guilt due to the absence of raising children, absence of a support network and anticipated suffering about life outside. of prison in the face of stigma and discrimination from society that does not allow job prospects outside the prison context^(18,19)^.

Finally, given the situation of vulnerability experienced by women in prison and the right to exercise motherhood, the perspective of Protection Bioethics^(21,22)^ enhances the need to implement the National Policy for Comprehensive Health Care for people deprived of liberty in the system prison (PNAISP) that aims to be legitimately moral, equitable and that respects and promotes human dignity^(23)^, as it proposes a space for the coexistence of mother and child who live in this context.

The category entitled “Mothers and children in prison exacerbating imbalances, tensions and conflicts” expresses the relevance of the discussion about the problems and different forms of violence, which multiply in prison. A study, when addressing female incarceration, highlighted numerous violations and constraints on the exercise of motherhood that were caused by disciplinary practices and situations of insecurity experienced by women behind prison walls^(24)^.

This population invisible to society, in general, experiences several intramural conflicts. A study carried out in four Brazilian states with 22 women, pregnant women and mothers with children in prison and 19 professionals showed that the disciplinary mechanisms characteristic of the penitentiary, when integrated with the control and surveillance practices of incarcerated women-mothers, specifically leave these women vulnerable, and expose their children to psychological and moral suffering^(5)^.

The relationships established within the prison context are influenced by the atmosphere of tension and insecurity that surrounds the prison environment. In this sense, the prison officer is the professional responsible for monitoring the prisoner, as well as her child, generating constant situations of tension and conflicts^(25)^.

It was observed in this study that prison officers also experience situations of vulnerability^(21)^, expressed by excessive workload and exposure to dangerous situations, which can promote psychological illness.

The agent still finds himself in the middle of a duality of hierarchy, configuring a paradox in which, on the one hand, he obeys the orders of his superiors and, on the other, he imposes them on the inmates^(26)^. Establishing a power relationship between the prison officer and the woman in custody. In this sense, research described that relationships with administration were the main cause for the development of stress in prison officers^(27)^. This group is one of those that most suffer from psychological illness. The activities carried out by prison officers are exhausting, both physically and emotionally, therefore, studies investigating aspects related to the health of these professionals are necessary^(28)^.

The interviewees’ speech revealed the existence of conflicts between the inmates themselves due to the delimitation of space, that is, territories of power within the prison and due to the divergence of criminal factions in the female penal complex. These circumstances further expose this woman to situations of vulnerability^(9,21)^ and point to the need to implement resocialization actions that are effective within the walls, such as school and professional workshops. Still, historically, the most feared problem in prison is rebellion, constituting a trigger for an aggressive, violent, punitive and exclusionary system.

The third category called “Limits and references for resocialization” made it possible to analyze the context of the resocialization of the person in custody. However, this study revealed potential risks to resocialization, as prison has reproduced a meaningless daily life marked by the carelessness of women deprived of liberty^(29)^.

It was observed that recidivism was recurrent among those in custody. The lack of intramural incentive, manifested by the absence of activities aimed at the resocialization of this sentenced woman, associated with the lack of opportunities, the situation of poverty experienced, leads to a cycle of recidivism in the Brazilian prison system.

A study on incarceration, work and resocialization in the São Paulo Women’s Resocialization Centers revealed that in addition to the remission of the sentence, it was observed that some women want to work in prison to occupy the time that is idle, to have some money to buy something they need. for themselves or their children and for having the possibility, even if distant, of entering the job market when they are free^(30)^.

Contrasting women’s expectations with the dream of social reintegration, the speeches of prison officers and health professionals signal disbelief in relation to resocialization in Brazil’s prison system. In this sense, in general, discourses predominate that delegitimize the proposal of resocialization and, therefore, do not favor situations that provide these women with the opportunity to enter the job market upon leaving prison.

National studies on the resocialization of incarcerated women have shown that even with the implementation of resocialization through work projects, there are still several obstacles for women leaving the prison system to obtain formal employment after their release, and this contributes to a vicious cycle that leaves women prone to returning to crime^(31-33)^. And, international research describes the difficulties faced by prisoners in viewing prison as a legitimate space for resocialization, due to the constant situations of humiliation, violence and degradation they experience in prison spaces^(34,35)^.

Therefore, the proposal of Protection Bioethics^(8,9)^ is a valid tool to evaluate the moral conflicts that permeate the experience of motherhood in prison within the scope of public health. Therefore, it aims to protect vulnerable groups with a view to their survival with dignity in the prison context.

### Study limitations

This research was limited to studying just two women’s prison units that had a nursery to accommodate children in the state of Bahia. It is believed, however, that the results presented here can contribute to subsidizing public policies aimed at the population deprived of liberty.

### Contributions to the field of Nursing

The study contributes to nursing, since these professionals will be able to restructure their strategies and actions with more appropriate and humanized care for the health of women and children in the context of prison, in an attempt to consolidate the PNAISP, since these women and Their children are exposed to situations of risk and damage to their health, making them more vulnerable to illness, which contributes to the increase in maternal and child morbidity and mortality rates in the country and around the world.

## FINAL CONSIDERATIONS

This study sought to give a voice to women deprived of liberty and health professionals and prison officers who experienced the phenomenon of motherhood in prison. This population is highly vulnerable, whether because they are sentenced by the courts and serve their sentence in a closed regime in the women’s prison unit, or because they work in the prison system, an environment that increases vulnerability.

It is noteworthy that the abuse suffered in prison interferes with individuals’ choices and has impacts on their lives, from the failure to achieve their life project to the implications for their physical and emotional health.

Given this scenario and considering the moral problems experienced by mothers deprived of liberty and their children, by health professionals and prison officers, the Bioethics of Protection proposal emerges as a valid tool to discuss ways of confronting these issues in the field of health. public in Brazilian prison units.

Therefore, Bioethics of Protection can be considered a framework capable of promoting a practice of support for vulnerable groups. Through reflection on the process of violation and through visibility, it potentially helps to promote the health and dignity of women in situations of deprivation of liberty.

Bioethics of Protection directs ways of looking at and reflecting on resocialization in prison and envisions the implementation of public policies that effectively reach this population. In relation to health professionals and prison officers, it is necessary to develop a policy of care for professionals who work in the prison system and to carry out psychological support for these individuals who work in this intensely stressful environment.

## References

[1] Ministério da Justiça e Segurança Pública (BR), Departamento Penitenciário Nacional, Divisão de Atenção às Mulheres e grupos específicos (2020). Mapeamento de mulheres presas grávidas, parturientes, mães de crianças até 12 anos, idosas ou doentes.

[2] Ferreira ACR, Rouberte ESC, Nogueira DMC, Maia RS, Costa EC. (2021). Maternal care in a prison environment: representation by story drawing. Rev Enferm UERJ.

[3] Ministério da Justiça (BR) (2014). Portaria Ministerial n. 210, de 16 de janeiro de 2014. Política Nacional de Atenção às Mulheres em Situação de Privação de Liberdade e Egressos do Sistema Prisional, e dá outras providências.

[4] Félix RS, França DJR, Nunes JT, Cunha ICBC, Davim RMB, Pereira JB. (2017). The nurse in pré-natal care for women in prison system. Rev Enferm UFPE.

[5] Aguilar A, Alexia R. (2021). Living conditions of children who grow up with their mothers in the prison farm of Izalco and in its annex. Masferrer Investiga.

[6] Cross J. (2020). Imprisoning pregnant and parenting women: a focus on social justice, equal rights and equality. Health Soc Work.

[7] Andrade ABCA, Gonçalves MJF. (2018). Motherhood in prison regime: maternal and neonatal. Rev Enferm UFPE.

[8] Schramm FR. (2008). Bioethics of Protection: a valid tool to face moral problems in the era of globalization. Rev Bioét.

[9] Schramm FR. (2009). The bioethics of the vulnerable [Entrevista].

[10] Geertz C. (2008). Uma descrição densa: por uma teoria interpretativa da cultura.

[11] Goshin LS, Arditti JA, Dallaire DH, Shlafer RJ, Hollihan A. (2017). An international human rights perspective on maternal criminal justice involvement in the United States. Psychol, Public Pol Law.

[12] Sykes GM. (1999). The society of captives: a study of a maximum prison.

[13] Moore H., Minaker J, Hogeveen B. (2015). Criminalized mothers, criminalizing mothering.

[14] Perusolo A, Oliveira MR, Pugliesi F, Dittrich MG. (2017). Uma etnografia num presídio feminino no sul do Brasil: mulheres-mães e seus filhos no cárcere. RBTS.

[15] Bardin L. (2011). Análise de conteúdo.

[16] Soares IR, Cenci CMB, Oliveira LRF. (2016). Mothers in prision: link perception with sons. Estud Pesqui Psicol.

[17] Pyrrho M, Schramm FR. (2019). Interfaces between collective health and bioethics: nanotechnology as an object-model. Rev Bioét.

[18] Mignon S. (2016). Health Issues of Incarcerated Women in the United States. Cien Saude Colet.

[19] Santos DSS, Bispo TCF. (2018). Mãe e filho no cárcere: uma revisão sistemática. Rev Baiana Enferm.

[20] Fries B, Schmorrow A, Lang SW, Margolis PM, Heany J, Brown GP (2013). Symptoms and treatment of mental illness among prisoners: a study of Michigan State prisons. Int J Law Psychiatr.

[21] Schramm FR. (2017). Bioethics of protection: a health practice evaluation tool?. Ciên Saúde Coletiva.

[22] Schramm FR. (2019). Public health: biotechnoscience, biopolitics, and bioethics. Saúde Debate.

[23] Araujo E, Schramm FR. (2017). Principles of a street clinic and the functioning of crack cocaine. Rev Bioet.

[24] Arinde EL, Mendonça MH. (2019). Prison policy and guarantee of comprehensive care to the health of the child who lives together with mother deprived of freedom, Mozambique. Saúde Debate.

[25] Santos DSS, Camargo CL, Bispo TCF. (2021). Child care in the prison setting: a view from the prison staff. Braz J Health Rev.

[26] Lourenço AS. (2011). O espaço de vida do agente de segurança penitenciária no cárcere: entre gaiolas, ratoeiras e aquários.

[27] Lima EMM, Soares IP, Santos ACM, Souza DO. (2018). Health of penitentiary agents in the Brazilian context. Rev Enferm UFPE.

[28] Akbari J, Akbari R, Shakerian M, Mahaki B. (2017). Job demand-control and job stress at work: a cross-sectional study among prison staff. J Educ Health Promot.

[29] Varella D. (2012). Carcereiros.

[30] Massaro CM., Lourenço LC, Gomes GLR. (2013). Prisões e punições no Brasil contemporâneo.

[31] Souza MS, Costa ASM, Lopes BC. (2019). Women and the production of the delinquent subject. Cad EBAPE.

[32] Lima RS. (2018). Violence and public safety as a democratic simulacrum in Brazil. Int J Criminol Sociol.

[33] Oliveira KRV, Santos AAP, Vieira MJO, Pimentel E, Comassetto I. (2020). Women prison inmates’ perceptions of access to health as a tool for resocialization. Rev Enferm UERJ.

[34] Golembeski CA, Sufrin CB, Williams B, Bedell PS, Glied AS (2020). Improving health equity for women involved in the criminal legal system. Womens Health Iss.

[35] Sufrin C, Beal L, Clarke J, Jones R, Mosher WD. (2019). Pregnancy Outcomes in US Prisons, 2016-2017. Am J Public Health.

