# Supplementary material for: Maternity behind and beyond bars: analysis from the perspective of protection bioethics
Source: Rev Bras Enferm. 2025 Jan 10;77(Suppl 4):e20220576. doi: 10.1590/0034-7167-2022-0576 (PMC11726811; doi:10.1590/0034-7167-2022-0576)
Supplement: Translation

# Translation: Maternidade atrás e além das grades: análise na perspectiva da bioética da proteção

**DOI:** 10.1590/0034-7167-2022-0576pt

**Authors:** Denise Santana Silva dos Santos, Dulcinéia Ghizoni Schneider, Mara Ambrosina de Oliveira Vargas

**Affiliations:** I Universidade do Estado da Bahia. Salvador, Bahia, Brasil; II Universidade Federal de Santa Catarina. Florianópolis, Santa Catarina, Brasil

**Keywords:** Prisões, Maternidade, Bioética da Proteção, Vulnerabilidade, Mulheres

## Abstract

**Objetivo:**

analisar de que modo se expressa a maternidade nas unidades prisionais femininas na perspectiva da Bioética da Proteção.

**Método:**

pesquisa qualitativa com abordagem etnográfica, desenvolvida em duas unidades prisionais femininas. Participaram do estudo seis mães em privação de liberdade; 15 profissionais de saúde e nove agentes penitenciárias. Para a coleta de dados, utilizou-se entrevista semiestruturada e observação descritiva. A Análise dos dados baseou-se na técnica de Análise de Conteúdo, categoria temática.

**Resultados:**

emergiram três categorias: mulheres e crianças vulneradas atrás das grades (iniquidades); mães e crianças no cárcere exacerbando desequilíbrios, tensões e conflitos; e limites e referências para a ressocialização.

**Considerações Finais:**

a proposta da Bioética da Proteção surge como ferramenta válida para o direcionamento analítico do processo de enfrentamento às questões no âmbito da saúde pública nas unidades prisionais, considerando os grupos vulnerados e visando à equidade e à dignidade humana.

## INTRODUÇÃO

O número de mulheres que se encontram no cárcere tem aumentado em todo o mundo nas últimas décadas, de forma que o Brasil possui a terceira posição no ranking mundial com 773.151 mil pessoas encarceradas sendo 37,2 mil mulheres. Conforme o mapeamento realizado pelo Departamento Penitenciário Nacional (DEPEN), no sistema prisional brasileiro em março de 2020, do total de mulheres presas, 12.821 eram mães de crianças de até 12 anos e 208 estavam grávidas^(1)^. Tal fato repercute no quantitativo de mulheres que vivenciam a maternidade no contexto do sistema prisional e, consequentemente, eleva o número de crianças que nascem no cárcere, tornando-as uma população vulnerável^(2)^.

A Política Nacional de Atenção às Mulheres em Situação de Privação de Liberdade e Egressas do Sistema Prisional estabelece condições mínimas de assistência às mães presas e aos recém-nascidos, uma vez que as leis e políticas públicas são de total relevância para minimizar os altos índices de morbimortalidade materna e infantil^(3)^. No entanto, o estudo evidenciou que apesar de existirem políticas públicas voltadas para as mulheres encarceradas, ainda estão distantes de ter seus direitos assegurados, respeitados e garantidos por lei^(4)^.

Essas mulheres/gestantes/mães encarceradas enfrentam situações de tensões, sofrimento, violência, insegurança, medo, ansiedade e impotência diante do dever de proteger a criança, da limitação de apoio socioafetivo, bem como da incerteza do reflexo do ambiente carcerário na vida do filho, já que as instituições não dispõem de infraestrutura para atender as necessidades do binômio^(5,6)^.

Ressalta-se, ainda, a exposição às condições vulneráveis de saúde e a fragilidade, comprometendo a saúde do binômio mãe-filho, desencadeando complicações como partos prematuros e baixo peso ao nascer, elevando, assim, os índices de morbimortalidade materna e neonatal^(7)^.

Apesar da existência de estudos que retratam o cotidiano do encarceramento feminino, há uma lacuna oriunda de pesquisas de campo que tratam a maternidade na prisão na perspectiva da Bioética da Proteção.

Dessa forma, refletir sobre os conceitos trazidos pela Bioética da Proteção, proposta formulada por pesquisadores latino-americanos, pode evidenciar conteúdos que decorrem da ética e estão relacionados aos problemas morais envolvidos pela vulnerabilização humana, aos danos e carências concretas^(8)^. Portanto, a Bioética da Proteção foi utilizada como instrumento direcionador para avaliar os conflitos morais que permeiam a vivência da maternidade no cárcere. Nessa perspectiva, este estudo adotou o conceito de vulnerado descrito por Schramm^(8,9)^ como pessoas que, por alguma razão independente de suas vontades, não são capazes de se defenderem sozinhas dadas as condições desfavoráveis em que vivem ou devido ao abandono das instituições vigentes que não lhes oferecem o suporte necessário para enfrentar sua condição de afetados(as) e tentar sair dela.

Sendo assim, a relevância deste estudo versa na reflexão sobre a situação de vulnerabilidade vivenciada pela mulher durante a maternidade no sistema prisional. A maternidade é compreendida como um fenômeno social e possui diversos significados de acordo com o contexto histórico^(4,7)^. Portanto, destaca-se que as condições de gestação, parto e nascimento influenciam na morbimortalidade materna e infantil, indicador que repercute no nível de desenvolvimento humano de um país.

## OBJETIVO

Analisar de que modo se expressa a maternidade nas unidades prisionais femininas, na perspectiva da Bioética da Proteção.

## MÉTODOS

### Aspectos Éticos

Esta pesquisa respeitou as exigências nacionais e internacionais regulamentadas pelas normas de pesquisa que envolvem seres humanos, sendo submetida e aprovada pelo Comitê de Ética em Pesquisa da Escola de Enfermagem da Universidade Federal da Bahia. Além disso, ela foi autorizada pela Secretaria de Administração Penitenciária e Ressocialização do Estado da Bahia (SEAP), sendo elaborado um termo de Consentimento Livre e Esclarecido (TCLE), que foi lido e assinado pelas participantes.

### Tipo de pesquisa e referencial teórico

Trata-se de um estudo qualitativo, descritivo de natureza etnográfica. Na etnografia, o ser social constrói e, muitas vezes, mantém em seu *modus vivendi*, seus costumes, tradições e crenças, de modo a perpetuá-los a cada geração^(10)^.

A etnografia tem sido utilizada em estudos internacionais^(11-13)^ e nacionais^(7,14)^, descrevendo a situação da população privada de liberdade, o que demonstra a pertinência dessa metodologia na apreensão do cotidiano, valores e culturas desenvolvidos dentro das unidades prisionais.

### Procedimentos metodológicos

#### Cenário do estudo

O lócus de pesquisa foi o Conjunto Penal Feminino de Salvador e a Unidade Prisional Feminina do Conjunto Penal de Feira de Santana, no estado da Bahia. A escolha por essas unidades se deu por possuírem berçário para acolher mãe e filho no referido estado.

A aproximação inicial com o campo de estudo e com as participantes aconteceu a partir dos Projetos de Iniciação Científica e de Extensão Universitária que desenvolvem ações de educação e de saúde com as mulheres e seus filhos na unidade prisional, por meio de oficinas mensais.

#### Fonte de Dados

Participaram do estudo seis mães que vivenciaram a maternidade em privação de liberdade; 15 profissionais de saúde (assistente social, enfermeiro, médico, nutricionista, psicólogo e técnico de enfermagem) ligados diretamente com o processo de cuidar de mães e seus filhos na prisão; além de nove agentes penitenciárias, totalizando, assim, 30 participantes.

Os critérios de inclusão da pesquisa foram: mães que vivenciaram a maternidade em privação de liberdade e estivessem cumprindo pena em regime fechado; agentes penitenciários e profissionais de saúde que compõem o quadro de funcionários da instituição e estiverem de plantão no momento da entrevista.

Foram excluídas da pesquisa as mães em privação de liberdade que estavam no regime semiaberto, ou que apresentaram alterações cognitivas. E agentes penitenciárias e profissionais de saúde que estavam de licença médica, portanto, afastados das suas atividades laborais.

O convite, para as participantes, foi realizado pela primeira autora, durante as atividades de extensão do projeto da unidade prisional. Após o aceite, foram agendados data, local e horário das entrevistas, conforme disponibilidade das participantes.

#### Coleta e organização dos dados

A coleta de dados seguiu com base nos critérios consolidados para relatar pesquisa qualitativa (COREQ) e ocorreu entre os meses de janeiro a julho de 2019, em duas etapas: a primeira foi documental, realizada por meio da análise dos prontuários e processos judiciais das mulheres/mães colaboradoras que estiveram nas unidades prisionais e registro dos dados em formulário; já a segunda etapa utilizou a entrevista semiestruturada e a observação descritiva.

As entrevistas foram realizadas individualmente, na sala do setor administrativo das unidades prisionais, gravadas por um gravador de voz digital (com consentimento prévio dos(as) participantes) e tiveram duração média de 30 minutos. Posteriormente, as entrevistas passaram pelo processo de transcrição, sendo essa etapa realizada por três bolsistas do Grupo Crescer, visto que houve um grande volume de dados. Ademais, todas as transcrições foram revisadas pela pesquisadora.

Depois de transcritas, as entrevistas foram codificadas com uma letra para caracterização dos participantes, quais sejam: mãe (M), profissional de saúde (PS), e agente penitenciária (AP). Junto à descrição pelas referidas iniciais, seguiu-se o número relativo à ordem em que foram realizadas, de forma a manter o anonimato dos(as) entrevistados(as).

#### Análise dos dados

Os dados obtidos foram analisados segundo a Análise de Conteúdo^(15)^, na modalidade Análise Temática. Para a análise, realizou-se o recorte das falas, levando-se em consideração a frequência dos temas extraídos das entrevistas, a fim de encontrar os principais núcleos de sentido, cuja presença dá significado ao objetivo proposto. Em seguida, iniciou-se um diálogo entre o material encontrado e os pressupostos teóricos escolhidos para a pesquisa. As informações coletadas no formulário foram trianguladas com os dados obtidos por meio das entrevistas semiestruturadas e as referências da literatura.

Assim, foram identificados três núcleos temáticos que sustentaram o conhecimento obtido neste estudo: (1) Mulheres e crianças vulneradas atrás das grades (iniquidades); (2) Mães e crianças no cárcere exacerbando desequilíbrios, tensões e conflitos e (3) Limites e referências para a ressocialização.

## RESULTADOS

### Caracterização dos participantes

Na descrição sociodemográfica, a pesquisa revelou que as participantes eram majoritariamente mulheres, apenas um homem foi entrevistado e a faixa etária variou de 18 a 55 anos.

Dentre as mulheres privadas de liberdade, observou-se que, em relação à escolaridade, cinco mulheres tinham ensino fundamental incompleto, sendo que apenas uma tinha o ensino fundamental completo. Quanto à condição conjugal, todas eram casadas ou viviam em união estável. Quanto à raça/cor, todas se autodeclararam negras (preta e parda).

Foi observado que entre os profissionais de saúde 11 eram negros e quatro eram brancos, sete referiram ser solteiras, sete casadas e uma em união estável. No que tange à profissão: três assistentes sociais, cinco enfermeiras, duas nutricionistas, uma médica, três psicólogas e uma técnica de enfermagem.

Dentre as agentes penitenciárias, quatro possuíam nível superior em cursos voltados para área da educação e uma das participantes possuía especialização. No que diz respeito ao tempo de atuação no sistema prisional, três tinham menos de 10 anos, duas tinham entre 10 e 20 anos e quatro tinham mais de 20 anos de serviço. Todas eram concursadas.

A partir da leitura, exploração e análise das falas, emergiram três categorias analíticas intituladas: “Mulheres e crianças vulneradas atrás das grades (iniquidades)”; “Mães e crianças no cárcere exacerbando desequilíbrios, tensões e conflitos” e “Limites e referências para ressocialização”.

#### (1) Mulheres e crianças vulneradas atrás das grades (iniquidades)

Na primeira categoria, denominada “Mulheres e crianças vulneradas atrás das grades (iniquidades)”, os discursos das participantes descreveram situações de violência vivenciadas pela mulher privada de liberdade:

*Minha vida foi muito difícil, meu companheiro me batia muito, tinha ciúme de tudo, saí de casa e fui morar com ele. Muita gente nem entendia porque eu ainda estava com ele, ele usava droga, toda vez que ele me via conversando com alguém, tudo era motivo para eu apanhar, se não quisesse ter relação sexual com ele, ele me batia. Quando eu vim presa ele me mandou um recado que se eu não tivesse o encontro íntimo com ele, ele me matava aqui na prisão, tinha medo dele, fui ameaçada de morte, várias vezes, por ele.* (M3)

A mulher no sistema prisional vivencia a solidão e a falta de rede de apoio para a realização dos cuidados com a criança.

*Nunca recebi visita de ninguém, nem de minha mãe, que por causa da minha prisão pegou dois empréstimos e agora ganha bem pouquinho e ainda cuida dos meus filhos. Tem horas que acho que vou enlouquecer de tanta saudade* [lágrimas nos olhos] ... [silêncio]. (M2)

*As mulheres são abandonadas no cárcere, elas são mais fiéis e não abandonam seu marido. Isso é um complicador para a mulher, pois aqui ela não tem o parceiro para ajudar no cuidado da criança.* (PS9)

A estrutura inadequada do ambiente prisional gera situações de iniquidade que interferem na prestação dos cuidados à criança. Os discursos das participantes expressam essa inadequação:

*Bem, aqui na prisão o ambiente é ruim. Dava o banho do menino na cela mesmo, a gente lava roupa também na cela e coloca as roupas para estender no pátio.* (M1)

*O principal desafio para mim é a infraestrutura das unidades prisionais para as crianças. Expor ela a um ambiente insalubre é prejudicial para sua vida.* (PS10)

Foi observado que o contexto de vulneração vivenciado pela mulher e seu filho na prisão desencadeia o adoecimento da criança.

*Meu filho aqui teve assadura, pois aqui é muito calor, ele ficava também de brotoeja. Depois ficou com alergia, tinha dificuldade de respirar por causa da fumaça daqui do pavilhão, aí tinha que chamar a agente penitenciária para levar ele para o médico avaliar, depois a gente era levado para a emergência para ele fazer a nebulização.* (M6)

*Com relação à doença, aqui no conjunto penal é muito quente na cela e quase não é disponibilizada fralda pela administração, quando a família e a Pastoral do cárcere não fornecem é bem complicado, aí as crianças terminam tendo assadura. Todas as crianças tiveram algum problema na pele, tipo assim brotoeja, coceira e assadura.* (PS1)

Constatou-se, também, a privação do contato social vivenciado pela criança que fica com sua mãe na cela sem interagir com outras crianças, familiares e parentes, predispondo a criança a futuros danos psíquicos.

[…] *e outra coisa também que mexe comigo é a questão que dói, a criança é inocente e já nasce preso, não tem um contato com brinquedo, com cores, com os outros parentes, com outras crianças, fica aqui nesse ambiente insalubre, com mulheres cumprindo penas relacionadas a diversas coisas, é um perigo. Ela não recebe visita de outras crianças, nem dos vizinhos, malmente vem a avó visitar, a mulher fica sozinha com a criança aqui dentro. (*AP 8)

*Para mim, o maior desafio de ter uma criança em privação de liberdade são os danos psíquicos que essa criança irá ter, é impossível sair desse inferno sem nenhuma sequela. Por isso, acho que não é legal a criança estar aqui, ela fica privada de contato social da interação com outras crianças.* (PS 2)

#### (2) Mães e crianças no cárcere exacerbando desequilíbrios, tensões e conflitos

Na segunda categoria denominada “Mães e crianças no cárcere exacerbando desequilíbrios, tensões e conflitos”, os discursos das participantes revelaram os conflitos vivenciados pela mulher na prisão e destacaram a relação conflituosa entre as internas e as agentes prisionais.

*Quando a gente se sente mal aqui é uma barra, muito difícil tudo, imagine para a criança, pois as prezadas* [agentes penitenciárias] *sempre acha que nós estamos mentindo, aí elas não acredita na gente e demora de vim, quando vem leva para a central médica e lá dá o remédio de febre, as pomadas para assadura ou brotoeja e pronto.* (M1)

Outro conflito sinalizado pelas entrevistadas revelou as brigas entre as próprias internas.

*Aqui no presídio está tendo muita confusão, muito conturbado, muitas brigas entre rivais. Ficava com medo dela aqui dentro e ter uma confusão e ela ser atingida.* (M5)

Os discursos das entrevistadas revelaram que o contexto prisional é marcado pela tensão, medo e pelo temor das rebeliões. Situações que deixam ainda mais vulneráveis as vivências da maternidade na prisão e expõem mulheres e seus filhos a situações de risco.

*Em questão da segurança, nossa função é afastar a criança daqueles grupos violentos, grupos mais agitados, que têm um clima de tensão maior. Então, a gente procura tirar desse grupo, afastar desse convívio, para que não venha afetar a saúde dela como um todo.* (AP 8)

*Minha maior dificuldade é a rebelião, tenho medo de ter um motim com bebês lá dentro, sabe, por quê? Se acontecer alguma coisa com esse bebê, a imprensa, a Defensoria Pública e todo mundo vai culpar para o resto da vida o setor de Segurança, dizendo que foi uma irresponsabilidade manter bebês lá dentro, e aí viu. Porque a criança é um ser incapaz e deve ser protegida.* (AP 4)

#### (3) Limites e referências para a ressocialização

Na terceira categoria denominada “Limites e referências para a ressocialização” emergiram nos discursos a situação da reincidência de mulheres no sistema prisional:

*Essa é minha segunda entrada na prisão, fui presa primeiro por tráfico de drogas* [8 anos de pena]*, fiquei foragida quando sai para um indulto de natal e não voltei, depois fui presa novamente por tráfico de drogas e já desci para o conjunto penal.* (M4)

*Assim, a minha primeira entrada na prisão estava grávida de Ester, fiquei 8 meses aqui e saí para Prisão Domiciliar fiquei 3 anos fora. Aí, na minha segunda entrada, retornei já estava grávida de Ruth no 8º mês de gestação fui direto para a cela da disciplina* [seguro] *onde fiquei 20 dias até Ruth nascer.* (M5)

Foi observada durante as entrevistas, a esperança na inserção no mercado de trabalho após o cumprimento da pena, evidenciando a necessidade da ressocialização da egressa do sistema prisional.

*É horrível estar longe do meu filho, não tenho palavras para decifrar, o que é estar longe dele. Tudo que eu mais quero é sair, arrumar um trabalho e cuidar do meu filho.* (M4)

Contudo, foi percebido também o descrédito no papel ressocializador da prisão por parte dos profissionais que trabalham no sistema prisional.

*Assim, não acredito na ressocialização, nem em nada que diz nesses manuais, para mim quem faz tem que pagar, é ladrão, roubou? Tem que pagar. Mas a criança não fez nada, ela só nasceu nesse lugar desgraçado, sem nenhuma condição de vida. Não tem referência de mãe, pois nossos pais são nossos heróis e ela* [criança] *não tem nada, está presa junto da mãe que é uma marginal e ainda colocou o filho aqui.* (PS2)

*Não acredito nas pessoas aqui, tipo assim que elas podem mudar? Se ressocializar? Não acredito nisso. Acredito que as pessoas não mudam e, por isso, as crianças são punidas junto com as mães.* (PS 5)

Por fim, foi evidenciada a função de proteção do Estado à mulher e seu filho custodiado no sistema prisional.

[…] *a gente quer proteger aquele ser inocente, o senso de proteção da mãe, queremos proteger o inocente que está sob a tutela do estado, a gente sofre de vê a criança nascer preso, de ficar tanto tempo e depois haver a separação.* (AP 8).

## DISCUSSÃO

O estudo evidenciou diversas situações de violência vivenciadas pelas mães e seus filhos nas unidades prisionais, revelando que essas mulheres são vulneradas, ou seja, são pessoas que não possuem os meios que as capacitam para realizar seus projetos de vida, portanto, elas não são capazes de alcançar uma vida digna^(8,9)^. Por esse motivo, estão expostas a uma série de riscos que tem implicações para sua saúde física e mental, bem como para a saúde de seu filho.

Essa situação é agravada pelas iniquidades existentes no sistema prisional, exemplificada em estudo^(6)^, o que sinaliza a inadequação da estrutura física, revelada através de celas úmidas e quentes, horários reduzidos para o banho de sol, ausência de local apropriado para o cuidado da criança.

Pesquisas^(5,7)^ revelam que a falta de estrutura dos presídios, além de afetar a ressocialização da mulher privada de liberdade, prejudica, sobretudo, a qualidade de vida dessas pessoas e de seus filhos. As precárias estruturas das prisões com relação às necessidades específicas das crianças, que vão desde a ausência de banheiros adequados para a higiene da criança até a carência e escassez de recursos, tais como fralda, roupa, pomada para prevenção da dermatite, falta de local adequado para estender as roupas da criança agravam as desigualdades e tornam piores as repercussões do encarceramento para essas mães e filhos.

Os resultados do presente estudo evidenciam as iniquidades em saúde existentes no contexto prisional, o que nos remete a pensar sobre as questões morais que acontecem rotineiramente e ao longo do tempo, dando visibilidade a carências e a injustiças sociais que não deveriam mais ocorrer. São realidades demonstradas pelos desequilíbrios sociais que determinam chances desiguais de acesso à saúde e a outros direitos fundamentais^(16)^.

Nesse sentido, as questões sociais vivenciadas pelos filhos de mães em privação de liberdade, podem, também, serem descritas como o isolamento social, com horários e dias para visita de familiares, sem oportunidade de ir para outros locais, brincar em um local adequado, a exemplo de praças e parques, interagir com outras crianças, condições fundamentais para a socialização da criança. No contexto de vulnerabilidade do cárcere, a criança fica confinada à cela juntamente com sua mãe^(17)^.

Tal contexto leva a criança ao adoecimento, pois expõe mães e filhos à doenças infectocontagiosas. Estudos^(17-19)^ apontam para altas taxas de doenças infecciosas. Isto é, 20% das mulheres nas prisões têm doenças infecciosas; 26,8% afirma realizar prática de atividade sexual de risco com vários parceiros; 11,5% apresentam doenças sexualmente transmissíveis; com alto risco de HIV/AIDS, hepatite C, bem como do papiloma vírus humano associado ao câncer cervical. Além disso, outras infecções sexualmente transmissíveis a que as mulheres correm maior risco de desenvolver quando entram na prisão são a clamídia, a gonorreia e a sífilis, associadas à vitimização sexual e à prostituição.

Os dados revelam a vulnerabilidade que essas mulheres/gestantes privadas de liberdade estão expostas, vivenciando situações de risco e danos à saúde, além de exporem a criança às mesmas condições, o que potencializa o risco de morbimortalidade neonatal. Muitas vezes, essas mulheres já vivenciaram situações de extrema vulnerabilidade na vida fora dos muros da prisão, perpetuando o cárcere, como também a exclusão social e as iniquidades em relação à saúde^(17,20)^.

Nesse sentido, os profissionais também estão expostos ao adoecimento, pela exposição a riscos psicossociais a riscos biológicos pelo contato com doenças transmissíveis. As condições de trabalho não são boas, pois a infraestrutura é precária, o ambiente é insalubre e os equipamentos e materiais são escassos, o que dificulta o desenvolvimento do trabalho de forma eficaz e segura^(18,20)^.

Outros aspectos abordados pelas entrevistadas foram a solidão e a falta de rede de apoio, elementos que potencializam a vulnerabilidade dessas mulheres privadas de liberdade. Historicamente, é sabido que as mulheres são abandonadas na prisão e experienciam a maternidade sozinhas. Pesquisas nacionais sinalizaram várias situações propulsoras de distúrbios mentais, sendo elas: solidão, tristeza pelo abandono dos familiares, medo pela inadequação na adaptação à cultura prisional, culpa pela ausência na criação dos filhos, ausência de rede de apoio e sofrimento antecipado sobre a vida fora da prisão frente ao estigma e à discriminação da sociedade que não possibilita perspectivas de trabalho fora do contexto prisional^(18,19)^.

Por fim, diante da situação de vulneração vivenciada pelas mulheres na prisão e face ao direito do exercício da maternidade, a perspectiva da Bioética da Proteção^(21,22)^ potencializa a necessidade de implementação da Política Nacional de Atenção Integral à Saúde das pessoas privadas de liberdade no sistema prisional (PNAISP), que visa ser legitimamente moral, equitativa e que respeite e promova a dignidade humana^(23)^, na medida em que propõe um espaço para o convívio de mãe e filho que vivem nesse contexto.

A categoria intitulada “Mães e crianças no cárcere exacerbando desequilíbrios, tensões e conflitos” expressa a relevância da discussão acerca dos problemas e as diferentes formas de violência, que se multiplicam na prisão. Um estudo, ao abordar o encarceramento feminino, evidenciou inúmeras violações e constrangimentos no exercício da maternidade, ocasionados por práticas disciplinares e situações de insegurança vivenciadas pelas mulheres atrás dos muros das prisões^(24)^.

Essa população invisível à sociedade, em geral, experiencia diversos conflitos intramuros. Realizado em quatro estados brasileiros, com 22 mulheres, gestantes, mães com filhos na prisão, além de 19 profissionais, um estudo evidenciou que os mecanismos disciplinares característicos da penitenciária, ao se integrar às práticas de controle e vigilância das mulheres-mães encarceradas, deixam vulneráveis, concretamente, tais mulheres e expõem seus filhos ao sofrimento psíquico e moral^(5)^.

As relações estabelecidas dentro do contexto prisional são influenciadas pelo ambiente de tensão e insegurança que circundam o referido espaço. Nesse sentido, a agente penitenciária é a profissional responsável pela vigilância da presa, bem como de seu filho, o que pode viabilizar situações constantes de tensão e conflitos^(25)^.

Foi observado, neste estudo, que as agentes penitenciárias também experienciam situações de vulneração^(21)^, expressas por carga de trabalho excessiva e pela exposição a situações de perigo, o que pode promover adoecimento psíquico.

O(a) agente encontra-se, ainda, em meio a uma dualidade de hierarquização, configurando um paradoxo em que, por um lado, obedece às ordens de seus superiores e, por outro, as impõe às detentas^(26)^. Sendo assim, estabelece-se uma relação de poder entre o agente penitenciário e a mulher custodiada. Nesse sentido, uma pesquisa descreveu que as relações com a administração foi a principal causa para desenvolvimento de estresse em agentes penitenciário^(27)^. Esse grupo é um dos que mais apresentam adoecimento psíquico. As atividades desenvolvidas pelos agentes penitenciários são desgastantes tanto fisicamente quanto emocionalmente, portanto, a elaboração de mãos estudos investigando aspectos relacionados à saúde desses profissionais faz-se necessário^(28)^.

Foi evidenciada, na fala das entrevistadas, a existência de conflitos entre as próprias internas por delimitação do espaço, ou seja, territórios de poder dentro da prisão, devido à divergência das facções criminosas no conjunto penal feminino. Essas circunstâncias expõem ainda mais essas mulheres a situações de vulneração^(9,21)^ e apontam para a necessidade de implementação de ações de ressocialização que sejam efetivas intramuros, tais como a escola e as oficinas profissionalizantes. Ainda, historicamente, o problema mais temido na prisão é a rebelião, constituindo-se num estopim de um sistema agressor, violento, punitivo e excludente.

A terceira categoria, denominada “Limites e referências para a ressocialização”, possibilitou analisar o contexto da ressocialização da custodiada. No entanto, este estudo revelou potenciais riscos à ressocialização, na medida em que a prisão tem reproduzido um cotidiano sem sentido marcado pelo descuido da mulher privada de liberdade^(29)^.

Observou-se que a reincidência era recorrente entre as custodiadas. A falta de incentivo intramuros, manifestada pela ausência de atividades que direcionam para a ressocialização dessa mulher sentenciada, associada à ausência de oportunidades e à situação de miséria, impele-as para um ciclo de reincidência no sistema prisional brasileiro.

Um estudo sobre encarceramento, trabalho e ressocialização nos Centros de Ressocialização femininos paulistas revelou que, para além da remissão da pena, houve a preferência de algumas mulheres em trabalhar na prisão, de modo a ocupar o tempo que fica ocioso, arrecadar algum dinheiro para comprar itens necessários para si ou para os filhos, além de representar a possibilidade, mesmo que distante, de se inserir no mercado de trabalho quando estiverem livres^(30)^.

Contrapondo a expectativa das mulheres pelo sonho da reinserção social, as falas das agentes penitenciárias e dos profissionais de saúde sinalizam um descrédito em relação à ressocialização no sistema prisional do Brasil. Nesse sentido, de modo geral, predominam discursos que deslegitimam a proposta da ressocialização e, portanto, não favorecem situações que oportunizem às mulheres a inserção no mercado de trabalho ao sair da prisão.

Estudos nacionais sobre a ressocialização de mulheres encarceradas evidenciaram que, mesmo com a implementação dos projetos de ressocialização pelo trabalho, ainda existem diversos obstáculos para que as egressas do sistema prisional consigam um emprego formal após sua libertação, isso contribui para a consolidação de um ciclo vicioso que deixa a mulher propensa a retornar para o crime^(31-33)^. Ainda, pesquisas internacionais descrevem as dificuldades das presas em visualizar a prisão como um espaço legítimo de ressocialização, devido às constantes situações de humilhação, violência e degradação que vivenciam nos espaços prisionais^(34,35)^.

Portanto, a proposta da Bioética de Proteção^(8,9)^ é uma ferramenta válida para avaliar os conflitos morais que permeiam a vivência da maternidade no cárcere no âmbito da saúde pública, pois ela visa proteger os grupos vulnerados visando a sua sobrevivência com dignidade no contexto prisional.

### Limitação do estudo

Essa pesquisa se limitou a estudar apenas duas unidades prisionais femininas que possuíam berçário para acolher as crianças no estado da Bahia. Acredita-se, porém, que os resultados aqui apresentados podem contribuir para subsidiar políticas públicas voltadas para a população privada de liberdade.

### Contribuições para a área da Enfermagem

O estudo contribui para a enfermagem, uma vez que esses profissionais poderão reestruturar suas estratégias e ações com um cuidado mais adequado e humanizado para a saúde da mulher e da criança no contexto do cárcere, na tentativa de consolidar a PNAISP, visto que essas mulheres e seus filhos estão expostos a situações de riscos e danos à saúde, tornando-as mais vulneráveis ao adoecimento, o que favorece a elevação dos índices de morbimortalidade materna e infantil no país e no mundo.

## CONSIDERAÇÕES FINAIS

Este estudo buscou dar voz às mulheres privadas de liberdade e aos profissionais de saúde e agentes penitenciárias que vivenciaram o fenômeno da maternidade na prisão. Essa população é altamente vulnerada, quer seja por estar sentenciada pela justiça e cumprir a pena em regime fechado na unidade prisional feminina, quer seja porque trabalham no sistema prisional, ambiente potencializador de vulnerações.

Destaca-se que a vulneração sofrida na prisão interfere nas escolhas dos indivíduos e impacta as suas vivências, desde a não realização do seu projeto de vida até as implicações na sua saúde física e emocional.

Diante desse cenário e considerando os problemas morais vivenciados pelas mães privadas de liberdade e seus filhos, pelos profissionais de saúde e agentes prisionais, a proposta da Bioética da Proteção surge como ferramenta válida para discorrer acerca de modos de enfrentamento a essas questões no âmbito da saúde pública nas unidades prisionais brasileiras.

Portanto, a Bioética da Proteção pode ser considerada um referencial capaz de promover uma prática de amparo a grupos vulnerados. Por meio da reflexão sobre o processo de vulneração e a partir da visibilidade, a Bioética da Proteção auxilia, potencialmente, a promoção da saúde e a dignidade da mulher em situação de privação de liberdade.

Tal conceito também direciona modos de olhar e de refletir sobre a ressocialização na prisão e vislumbra a implementação de políticas públicas que efetivamente alcancem essa população. Em relação aos profissionais de saúde e agentes penitenciários, faz-se necessária a elaboração de uma política de atenção voltadas para esse público e a realização do acompanhamento psicológico para os indivíduos que trabalham nesse ambiente de intensa tensão.
